# Physicochemical Properties of Dried and Powdered Pear Pomace

**DOI:** 10.3390/molecules29030742

**Published:** 2024-02-05

**Authors:** Anna Krajewska, Dariusz Dziki, Mustafa Abdullah Yilmaz, Fethi Ahmet Özdemir

**Affiliations:** 1 Department of Thermal Technology and Food Process Engineering, University of Life Sciences in Lublin, 20612 Lublin, Poland; anna.krajewska@up.lublin.pl; 2 Department of Analytical Chemistry, Faculty of Pharmacy, Dicle University Science and Technology Research and Application Center (DUBTAM), 21280 Diyarbakır, Türkiye; mustafaabdullahyilmaz@gmail.com; 3 Department of Molecular Biology and Genetics, Faculty of Science and Art, Bingöl University, 12000 Bingöl, Türkiye; ozdemirfethiahmet23@yahoo.com

**Keywords:** pear powder, fruit by-products, fruit waste, grinding, antioxidant activity, phenolic compounds, LC-MS/MS, freeze-drying, contact-drying, drying kinetics

## Abstract

Pear pomace, a byproduct of juice production, represents a valuable reservoir of bioactive compounds with potential health benefits for humans. This study aimed to evaluate the influence of drying method and temperature on pear pomace, specifically focusing on the drying kinetics, grinding characteristics, color, phenolic profile (LC-MS/MS), and antioxidant activities of the powder. Drying using the contact method at 40 °C with microwave assistance demonstrated the shortest duration, whereas freeze-drying was briefer compared to contact-drying without microwave assistance. Freeze-drying resulted in brighter and more easily comminuted pomace. Lyophilized samples also exhibited higher total phenolic compound levels compared to contact-dried ones, correlating with enhanced antioxidant activity. Twenty-one phenolic compounds were identified, with dominant acids being quinic, chlorogenic, and protocatechuic. Flavonoids, primarily isoquercitrin, and rutin, were also presented. Pear pomace dried via contact at 60 °C contained more quinic and protocatechuic acids, while freeze-dried pomace at the same temperature exhibited higher levels of chlorogenic acid, epicatechin, and catechin. The content of certain phenolic components, such as gallic acid and epicatechin, also varied depending on the applied drying temperature.

## 1. Introduction

Food and green wastes constitute nearly half of the global waste generated, making them the largest waste category. It is projected that their volume will continue to rise, which is associated with technological and economic advancement, as well as population growth and consumption patterns [1]. Among the primary waste products of the fruit industry is pomace, a by-product of juice extraction. The proportion of fruit pomace relative to the raw material utilized can range from 10% to 35%, depending on the processed fruit and the extraction technology employed [2]. Inadequate management of these by-products could contribute to environmental risks and the release of hazardous greenhouse gases [3,4]. From an economic standpoint, such mismanagement is also inefficient as it results in the loss of valuable nutrients and health-promoting components, particularly in the context of malnutrition [5]. It is estimated that only 20% of pomace finds useful applications, such as animal feed, organic fertilizer, or pectin extraction for ethanol production [6,7].

In recent years, there has been growing interest in utilizing fruit by-products in human nutrition, as they offer potential sources of ingredients such as fiber, phenolic compounds, vitamins, and minerals [8]. Due to their high water content and the abundance of active compounds, prompt processing is necessary, especially for medium- and long-term storage. Moisture reduction techniques, such as drying, preserve the physical and chemical properties of the raw material, prevent spoilage, and reduce transportation costs [9]. Incorporating fruit waste into food products may have a positive impact on public health by enhancing their nutritional value, and increasing fiber and polyphenolic intake [10]. Furthermore, fortification can extend the shelf life of products and enhance their oxidative stability, while maintaining or even improving sensory properties [8].

Pear pomace (PP), which includes the peel, pulp, stem, core, and seeds remaining after industrial juice production, is known to contain 44 to 79% fiber on a dry weight basis, as well as organic acids, triterpenes, and polyphenols [11,12]. Fresh PP can contain up to twice as many polyphenolic compounds as the whole fruit, with levels reaching up to 18 g·kg^−1^ of fresh weight [13]. Thus far, in PP, elevated concentrations of procyanidins have been documented, accompanied by the presence of hydroxycinnamic acids, particularly 5-caffeoylquinic acid. PP was also characterized by the presence of diverse organic acids, including malic acid, citric acid, and shikimic acid, alongside triterpenes such as oleanolic acid and ursolic acid. Furthermore, polymeric procyanidins and arbutin were identified as additional constituents in PP. Notably, anthocyanins, specifically cyanidin derivatives, exhibited exclusive localization within the peel of the fruit, while being absent in the pulp, seeds, and leaves [14]. The aqueous extract of PP exhibited potential protective effects and the ability to inhibit lipid peroxidation, as observed in a study involving rats fed a high-fat/cholesterol diet [12]. Moreover, PP demonstrated anti-obesity effects, as evidenced by its positive impact on glucose homeostasis and modulation of gut microbiota composition in mice [15,16]. The aforementioned studies strongly indicate that incorporating PP into food products as a means of enrichment is a promising and practical choice.

The drying conditions applied to fruit by-products significantly influence their grinding characteristics and quality, including factors such as color, activity, water content, chemical composition, and antioxidant properties [17,18]. However, there is a lack of information in the current literature regarding the effect of drying methods and temperatures specifically applied to PP on its grinding process and physicochemical properties, which, in turn, impact the food properties of the resulting product.

Therefore, this study aims to investigate the effects of contact-drying and freeze-drying at various hotplate temperatures on the drying kinetics, energy intensity of the pomace comminuting process, powder color, profile of phenolic compounds, as well as antioxidant activity.

## 2. Results and Discussion

### 2.1. Drying Kinetics and Fitting of the Drying Curves

**Figure 1:**
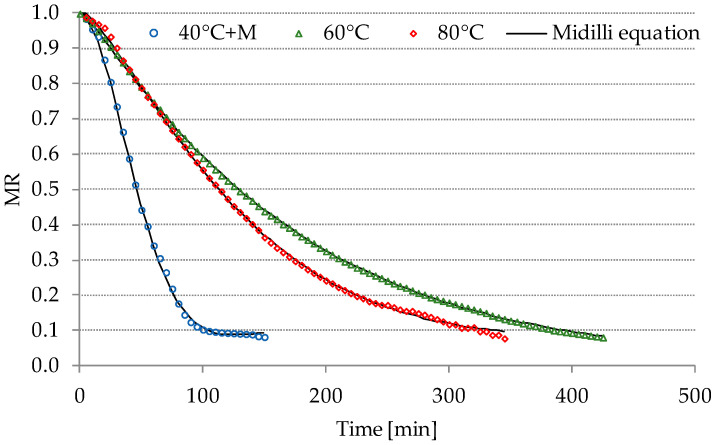
Drying curves of the contact-drying process for PP. MR—moisture ratio; M—microwaves 50 W.

**Figure 2:**
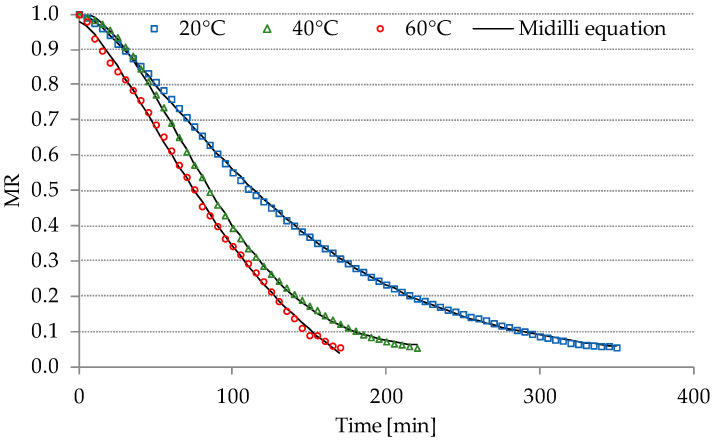
Drying curves of the freeze-drying process for PP. MR—moisture ratio.

The drying curves delineate alterations in the diminished moisture ratio (MR) relative to the elapsed duration of freeze-drying and contact-drying processes, as depicted in Figure 1 and Figure 2. Microwave-assisted contact-drying at 40 °C (40 °C+M) exhibited the shortest duration among the various drying methods, specifically requiring only 150 min. Notably, freeze-drying demonstrated a shorter duration compared to the contact-drying method at the same temperature of 60 °C. This observation may be attributed to the diminished pressure during freeze-drying, and higher temperature facilitating the sublimation of water. Many authors indicate that sublimation drying is a significantly longer process than air drying [19,20]. However, they do not consider sublimation drying at an elevated temperature, which, as demonstrated, significantly shortens the duration of the process. The adoption of freeze-drying at 60 °C, as opposed to contact-drying, resulted in a reduction of the drying time from 425 to 170 min. Moreover, an inverse relationship between the process time and the escalating temperatures in both contact-drying (ranging from 60 to 80 °C) and sublimation drying was evident. This trend aligns with the findings reported by Macedo et al. [21] regarding the convection drying of banana slices.

Regression analysis outcomes for seven distinct models, employed to expound the kinetics of freeze-drying and contact-drying, are delineated in Table 1 and Table 2. The coefficient of determination (R^2^) values, indicative of the efficacy of the drying methodologies across varied temperatures, exhibited a spectrum, spanning from 0.942 to 1.000. Concurrently, the root mean-square error (RMSE) manifested variations within the interval of 0.000 to 0.046. The coefficients of the equations describing the drying processes of PP are presented in Table 3 and Table 4.

Paramount fidelity to the experimental data was achieved through the Midilli model, substantiated by an R^2^ value attaining 1.000 at lower temperatures in both freeze-drying and contact-drying modalities, and registering 0.999 at 60 °C for freeze-drying and 80 °C for contact-drying. The preeminent RMSE recorded within this model was 0.001, affirming its optimal congruence with the empirical data. The Midilli model is one of the most often used and highest rated empirical models with freeze- and convective-drying properties, coherently demonstrated in various studies, including investigations on trappia peels and seeds [22], chokeberries [23], and onions [24]. 

### 2.2. Grinding Results

The particle size distribution of dried PP is provided in Table 5. Size reduction, a process intricately linked to chemical and microbiological stability, stands as one of the most pivotal and energy-intensive operations within the domain of the food industry [25]. In this context, we explored the effects of drying method and temperature on grinding energy requirements and particle size distribution of ground PP. Freeze-drying is noted for its capacity to effect a more pronounced reduction in particle size, rendering freeze-dried materials more amenable to fragmentation when compared to their air-dried counterparts [26]. This difference is attributed to the more porous and more brittle structure in freeze-dried materials [27]. It is worth noting that heightened hardness is recognized as a pivotal contributor to the generation of larger particle sizes following the grinding process, thereby leading to decreased process efficiency and increased energy consumption [28]. Based on the analysis of parameters d_10_, d_50_, and d_90_, it has been confirmed that lyophilization resulted in a more finely fragmented material of pear pomace compared to contact-drying. Samples freeze-dried at 60 °C boasted the highest concentration of fine particles, aligning with a d_10_ value of 39.2 µm. Conversely, contact-drying at 40 °C with microwaves yielded the lowest concentration, affirming a d_10_ value of 69.0 µm. PP freeze-dried at a plate temperature of 60 °C exhibited 39, 43, and 42% lower values for the d_10_, d_50_, and d_90_ parameters, respectively, compared to pear pomace dried by contact-drying at the same temperature. Moreover, the influence of drying temperatures was observed in the resultant degree of fineness. Increasing the drying temperature resulted in a reduction of the d_10_ parameter value in each of the employed methods. In the case of contact-drying, temperature did not affect particle size medians and the value of the d_90_ parameter. For lyophilizates, these values changed with temperature variation; however, a linear correlation was not observed. Moreover, the influence of the drying method and temperature on Span was negligible.

The assessment of technology profitability hinges upon the precise quantification of energy consumption. The current scientific literature presents a notable gap in terms of comprehensive data pertaining to the influence of various drying methods and temperatures on the energy requirements for grinding of PP. Our investigation has substantiated that the selection of the drying method significantly influences the energy demands of the grinding process. The grinding energy indices, moisture content, and water activity of PP are provided in the Table 6. Concretely, PP subjected to contact-drying demanded a significantly greater energy input for the grinding process when contrasted with pomace subjected to freeze-drying. The presence of elevated water content, observed in air-dried products in comparison to their freeze-dried counterparts, serves as a plasticizer between food materials, thus rendering them more resilient to comminution [28]. This discrepancy is also manifest in the variations observed in the grinding efficiency index. The specific grinding energy for freeze-dried PP ranged from 8.83 to 9.07 kJ·kg^−1^, whereas for contact-drying changed from 12.06 to 12.66 kJ·kg^−1^. In the case of kiwi fruit lyophilized under the same pressure but without the use of heating plates, the specific grinding energy was 13.6 kJ·kg^−1^ [29]. This discrepancy can be ascribed to the inherent characteristics of the respective raw materials, disparities in pre-drying processing, and the ultimate moisture content influenced by the utilization of varying drying temperatures. Remarkably, the temperature at which the drying process is conducted did not wield a substantial influence on energy consumption, regardless of whether lyophilization or contact-drying is employed. This observation aligns with the research conducted by Jewiarz et al. [30], which did not identify a robust correlation between air-drying temperatures and the energy demand for drying herbaceous and woody biomass materials at 60, 100, and 140 °C. Nonetheless, it is imperative to highlight an exception to this prevailing pattern. Within the scope of our study, lyophilized PP processed at 60 °C exhibited superior grinding efficiency when juxtaposed with other freeze-dried samples.

### 2.3. Color Coordinates 

Color is one of the essential attributes of food that impacts acceptability, recognizability, and consequently, the selection of the final product into which fruit pomace was incorporated [31,32]. Both the drying method and process parameters generally influenced the colorimetric parameters of PP. Lyophilized PP displayed a brighter color than those dried by the contact method. Nevertheless, no significant difference was observed in terms of L* values with respect to freeze-dried temperature. In the case of contact-drying, luminosity notably decreased during the drying process at 80 °C, compared to contact-drying at 40 °C with microwave assistance and at 60 °C (Table 7). This might have been attributed to the presence of oxygen during the process and material shrinkage [33]. In the study by Mrad et al. [34], convective drying temperature did not affect pear brightness. Nevertheless, the maximum temperature employed in that study was 70 °C, aligning with our findings, as the L* value of the PP only decreased when a temperature of 80 °C was applied during contact-drying. Moreover, the freeze-dried PP exhibited reduced redness compared to the contact-dried material. A decline in red hues was also noted upon the application of the highest temperature in both lyophilization and contact-drying. Freeze-drying of the pomace additionally led to a decrease in yellow tones, compared to the contact-drying method. Also, Yan et al. [33] found that the drying method of bitter gourd slices had a significant impact on the color of the dried product; consequently, sublimation-dried slices were also brighter, less red, and less yellow compared to hot air-dried ones. Nonetheless, lyophilization temperature did not substantially alter the b* value of PP. Similarly, no significant disparity was observed in redness for pomace dried by the contact method at 40 °C+M and 60 °C, as well as between 60 °C and 80 °C. 

### 2.4. Antioxidant Activity 

The Folin–Ciocalteu test is perceived as one of the measures of antioxidant capacity, and the total phenolics content (TPC) determined in our study was considered an approximate representation of the antioxidant substances content [35]. TPC in PP varied depending on the drying method and temperature, ranging from 2.58 to 4.32 mg of gallic acid equivalent (GAE)·g^−1^ DW (Table 8). Ferreira et al. [36] reported a slightly higher content of PP air-dried at 80–85 °C, ranging from 3.76 to 5.13 mg GAE·g^−1^ DW, depending on the extraction method and particle size. The higher phenolic content in the mentioned study may be attributed to the convective drying method, resulting in a shortened drying time of 110 min (compared to contact-drying at 80 °C in our study for 345 min). Additionally, result disparities emphasize the impact of extraction method and solvent choice on TPC.

Antioxidant activity of PP depended on the drying method. The lyophilized PP demonstrated the highest TPC, followed by contact-dried PP at 40 °C, and the lowest TPC was observed in contact-dried PP at 60 °C. Freeze-dried PP at 60 °C contained approximately 40% more phenolics compared to contact-dried pomace at the same temperature. These compound variations were reflected in the DPPH and ABTS radical scavenging activities; lyophilized extracts showed significantly lower EC_50_ values compared to air-dried pomace (Table 8). Conversely, Zhang et al. [37] found no difference in TPC between freeze-dried and hot-air-dried blueberry pomace at 50 °C, while TPC was lower at 40, 60, and 70 °C. In our study, the higher TPC suggests that elevated hot plate temperatures during lyophilization may be necessary to maximize TPC and AA in the final product. Prolonging drying time to 36 h negatively impacted the discussed compounds. Despite this, AA of lyophilized blueberry pomace against DPPH and ABTS was markedly stronger compared to the inhibitory activity of air-dried pomace. In our study, a significant correlation between TPC and EC_50_ in DPPH and ABTS inhibition was observed (r = −0.894, *p* = 0.016 and r = −0.962, *p* = 0.002, respectively). Furthermore, a significant correlation was found between the EC_50_ in the DPPH assay and the EC_50_ in the ABTS assay (r = 0.975, *p* = 0.001). Differences in correlation between our study and the cited research may arise from the specific phenolic profile changes during drying under distinct conditions. Phenolic compounds exhibit antioxidant activity associated with the number of available hydroxyl groups, and some may act synergistically, additively, or antagonistically [38].

The drying temperature also influenced TPC and AA of pear pomace but only in the case of contact-drying. Contact-dried samples at the lowest temperature with microwave assistance contained significantly more phenolics and exhibited higher antioxidant activity compared to those contact-dried at higher temperatures. However, with the increase in contact-drying temperature from 60 to 80 °C, the TPC and AA clearly increased. Comparing these results with drying time data at different temperatures, it can be concluded that reducing the drying process time (using microwaves or raising the temperature to 80 °C) positively affects TPC and AA of PP. Our findings align with Llavata et al. [39], who found that with the increase in convective drying temperature from 40 to 60 and then 80 °C, the TPC of apple pomace increased. In the cited study, TPC decreased with higher temperatures. Therefore, it can be speculated that 80 °C is the optimal temperature for air-drying fruit pomace if the goal is to maximize phenolic content. Interestingly, in the case of lyophilization, temperature generally did not affect TPC and AA of PP; only when the lowest temperature was applied, was the antioxidant effect more pronounced. 

### 2.5. Phenolic Profile of PP

The composition of phenolic compounds in PP, influenced by the drying method and conditions, is presented in Table 9. In total, 21 phenolic compounds from various phenolic families were identified. Phenolic acids constitute the main category of polyphenols in both immature and mature pears of different varieties [40,41]. The dominant polyphenolic acids in pear pomace were quinic acid and chlorogenic acid, consistent with the findings of Sun et al. [40]. They identified quinic acid as the predominant phenolic acid in ethanol extracts from fresh, immature pears, encompassing 10 different varieties, comprising approximately 73% of the total phenolic acid content. Furthermore, they confirmed that chlorogenic acid was the second most significant phenolic acid in pears, accounting for 24% of the total phenolic acid content. The third dominant phenolic acid in powdered PP was protocatechuic acid.

The drying method generally influenced the content of individual phenolic acids. Pear pomace subjected to contact-drying at 60 °C exhibited significantly higher levels of quinic acid compared to lyophilized samples at the same temperature. The content of protocatechuic acid was 2.6 times higher in pear pomace contact-dried at 60 °C compared to lyophilization carried out at the same temperature. Similar trends were observed in the case of black carrot pomace dried by convection at 65 °C, which contained twice as much protocatechuic acid compared to the freeze-dried counterparts [42]. Yuste et al. [43] noted that lyophilized apples lacked protocatechuic acid, while air-dried apples contained 14.6 mg·kg^−1^ DW of this compound. The lack of detection of this phenolic acid in the freeze-dried samples may be attributed to the lower process temperature compared to our experiment. Additionally, PP subjected to contact-drying contained trace amounts of salicylic acid, whereas lyophilized samples showed no detection of this compound.

On the other hand, freeze-dried PP at a heating plate temperature of 60 °C was richer in chlorogenic acid compared to pomace dried through contact-drying at the same temperature. Jiang [44] also observed a similar trend in the drying of Asian pear when comparing lyophilization (without heating plates) to air-drying (at 65 °C). Gallic acid was present in significantly higher amounts in lyophilized PP samples compared to pomace dried through contact-drying at 60 and 80 °C, while pomace dried through contact-drying with microwave assistance did not contain gallic acid at all. The results of Valadez-Carmona et al. [45] also showed that freeze-dried cacao pod husk contained approximately 28% more gallic acid than air-dried.

Both isoquercitrin and rutin are flavonoids, glycosides of quercetin, which exhibit antioxidant, anticancer, anti-inflammatory, and antidiabetic properties [46]. In this study, it was demonstrated that PP contained particularly high levels of isoquercitrin, ranging from 4.03 to 13.89 mg·g^−1^ DW. The content of rutin in PP ranged from 0.92 to 2.67 mg·g^−1^ DW, similar to air-dried grape pomace at 50 °C, with a content ranging from 1.13 to 2.84 mg·g^−1^ DW depending on the grape variety [47]. Hesperidin, vanillin, astragalin, and nicotiflorin were also present in PP but in significantly lower amounts. Additionally, trace amounts of naringenin were detected in all the analyzed samples.

Lyophilized PP exhibited noteworthy concentrations of epicatechin (ranging from 13.78 to 21.59 mg·g^−1^ DW) and limited quantities of catechin (from 0.3 to 0.43 mg·g^−1^ DW). Conversely, these compounds were undetectable in contact-dried pomace. Çoklar and Akbulut [48] identified a parallel pattern in their investigation, revealing an approximate two-fold increase in epicatechin and catechin levels in freeze-dried black grapes (excluding the use of hot plates) compared to oven-dried grapes at 60 °C. This implies that opting for lyophilization over convective drying may exert a beneficial influence on the concentrations of epicatechin and catechin.

The content of individual phenolic compounds also depended on the drying temperature. The quinic acid content significantly increased with the elevation of temperature to 60 and 80 °C in contact-dried powders, compared to those dried at 40 °C using microwaves. In the case of freeze-drying, the quinic acid content did not show a significant change under the influence of temperature modification. Furthermore, the content of gallic acid in pear pomace noticeably decreased with the temperature increase from 60 to 80 °C, whereas at 40 °C and with microwave application, its presence was not detected. These findings align with the report by Ghafoor et al. [49], which documented an approximate 13% decrease in the gallic acid content in contact-dried plums as the temperature increased from 60 to 80 °C. Similarly, in freeze-drying, the gallic acid content in PP significantly decreased with the increase in plate temperatures exceeding 20 °C.

The epicatechin content did not differ significantly in lyophilized samples at plate temperatures of 20 and 40 °C. Still, it increased at 60 °C, indicating that a higher drying temperature led to an increase in epicatechin content. Opposite trends were reported by Heras-Ramírez et al. [50] in convectively dried apple pomace, where the epicatechin content decreased significantly from 7.68 to 4.23 mg·100 g^−1^ DW with the increase in drying temperature from 50 to 80 °C. Interestingly, in contact-dried PP, epicatechin was not detected at all. The discrepancies may be attributed to the specific characteristics of individual raw materials and differences in both epicatechin content and other components affecting epicatechin stability during processing. Moreover, despite both contact and convective drying involving the flow of the drying agent, they differ in terms of duration and the mode of contact with the heat carrier. These differences could contribute to the degradation of epicatechin in the case of contact-drying. For other detected components in PP, no clear trend was observed between the content of individual polyphenolic substances and the increasing drying temperature.

## 3. Materials and Methods

### 3.1. Reagents and Raw Material

Gallic acid, methanol, ABTS (2,2′-azino-bis-(3-ethylbenzothiazoline-6-sulfonic acid), DPPH (1,1-diphenyl-2-picrylhydrazyl), sodium bicarbonate, potassium persulfate, Folin and Ciocalteu′s phenol reagent, and standard phenolic compounds (Table 9) of LC-MS/MS were analytical grade and were purchased from Sigma (Sigma-Aldrich GmbH, Steinheim, Germany). 

Full-ripe pears of the Konferencja variety were purchased from a local store and underwent a washing procedure. Subsequently, they were sliced, and their seed nests and stalks were removed. PP was extracted from the pears using a twin-screw juicer, Angel Juicer (Angel 5500, Angel Juicers, South Korea, Makpo) and then subjected to analysis. The part of raw material intended for freeze-drying was placed in a freezer chamber (GTL-4905, Liebherr, Sweden, Gothenburg) and frozen at −30 °C.

### 3.2. Drying Process

Freeze-drying (FD) and contact-drying (CD) experiments were conducted on PP. The FD process utilized an Alpha 1–4 Martin Christ freeze-dryer (Gefriertrocknungsanlagen GmbH in Osterode am Harz, Germany), employing a single-sided contact heat delivery method. Heating plates were subjected to three different temperatures (20 °C, 40 °C, and 60 °C with an accuracy of ± 2 °C) at a drying chamber pressure of 100 Pa. CD, more broadly referred to as air-drying, was conducted using a convection dryer (Promis-Tech, Wroclaw, Poland). The drying process involved placing a single layer of 100 g of the samples on trays exposed to an air velocity of 0.5 m/s (with an accuracy of ±0.1 m/s) at temperatures of 40 °C (with microwave assistance of 50 W), 60 °C, and 80 °C with an accuracy of ±1 °C). Microwaves were employed in CD, as a temperature of 40 °C did not yield a desiccated product suitable for proper pulverization. Contact-drying was chosen over convection drying due to the pomace’s pulp form, making it impractical to position the raw material for direct exposure to the drying agent. The FD and CD (100 g samples) procedures continued until the moisture content of the PP reached between 6 and 8%. During measurements taken every 5 min, changes in the mass of the dried material sample were constantly recorded. 

### 3.3. Modeling of Drying Curves

The kinetics of the process of drying was estimated using changes in the reduced water content (MR) as a basis:

(1) $$\mathrm{MR}=\frac{u_{t}}{u_{0}}$$

where u_t_ stands for the water content during the drying process (kg H_2_O·kg^−1^ DW) and u_0_ represents the initial water content (kg H_2_O·kg^−1^ DW). The equilibrium water content in this equation was disregarded because its value is insignificant in relation to u_0_ and u_t_. This kind of simplification is frequently employed and has little effect on the drying kinetics results [51].

To identify the most suitable mathematical model for capturing the kinetics of sublimation and convection drying of pear pomace, seven equations commonly cited in the literature were analyzed, as presented in Table 10.

### 3.4. Moisture Content and Water Activity

To assess the moisture content of PP, the gravimetric method was employed. Five gram samples were subjected to drying in a laboratory dryer at 105 °C until a constant weight was achieved. The water activity was measured using a water activity measurement device (LabMaster, Novasina, Swiss, Lachen).

### 3.5. Grinding Energy and Particle Size Analysis

PP was subjected to grinding for 60 s using a knife grinder (GRINDOMIX GM-200, Retsch, Germany, Haan, 1000 W, 10,000 rpm). For the measurement and computation of grinding energy, a computer system was integrated into the mills to monitor and assess the amount of energy expended during grinding [59]. The energy utilized in grinding was measured using a digital multimeter (VC870, Voltcraft^®^, Germany, Wollerau), connected to a computer running a program (VC870 Interface 4.2.6., Voltcraft^®^, Germany, Wollerau) that recorded the data every 1 min. 

The particle size distribution of PP powder was assessed through laser light scattering employing a laser particle size analyzer (Malvern Mastersizer 3000, Malvern Instruments Ltd., Worcestershire, UK). In laser diffraction measurement, a 5 g sample of fruit powder was automatically analyzed for particle size by passing a laser beam through it. The size dispersion index (Span) was determined using the following equation:

(9) $$Span=\frac{d_{90}-d_{10}}{d_{50}}$$

where d_10_, d_50_, and d_90_ represent diameters below which 10, 50, and 90% of the sample particles are smaller, respectively [60]. Each sample underwent three repetitions. 

The grinding efficiency index was calculated as the ratio between the surface area of the PP powder and the grinding energy. The specific grinding energy was determined by dividing the grinding energy by the mass of the PP powder [61].

### 3.6. Color Coordinates

The CIELab* system was employed to determine the color coordinates of PP using a colorimeter (NR20XE, Shenzhen Threenh Technology Co., Shenzhen, China). The system’s lightness, denoted as L*, ranges from 0 (perfect black body) to 100 (perfect white body), with a* coordinate signifying the shift from green (−a*) to red (a*), and b* coordinate representing the transition from blue (−b*) to yellow (b*) [62]. Hue angle was also calculated according to the equation:

(10) $$h^{*}=atan\frac{b^{*}}{a^{*}}$$

### 3.7. Antioxidant Capacity

#### 3.7.1. Preparation of Extracts

In total, 1000 mg of pear pomace powder was extracted for 30 min in a 5 mL methanol (100%):water (1:1, *v*/*v*) mixture, with stirring using a rotator (Multi Bio RS-24, Biosan Sia, Latvia, Riga). Subsequently, the samples were centrifuged for 5 min at 5000 rpm (3070× *g*) (LC8 3500, Benchmark, Sayreville, NJ, USA). The supernatant was collected, and the extraction procedure was repeated two more times. The resulting extracts were combined and stored in darkness at 20 °C.

#### 3.7.2. Total Phenolics Content (TPC) 

TPC was determined using the modified Folin–Ciocalteu method [63]. Specifically, 0.1 mL of the extract was mixed with 0.1 mL of distilled water and 0.4 mL of the diluted Folin reagent (with water 1:5, *v*/*v*). After 3 min, 2 mL of 7% sodium carbonate was added to the mixture, followed by vigorous shaking for 1 min. After incubating for 30 min in a dark environment, absorbance was measured at 760 nm. TPC was quantified in milligrams per gram of dry weight as gallic acid equivalents (y = 0.0039x + 0.0142, R^2^ = 0.9941). The blank sample was prepared using a methanol:water (1:1, *v*/*v*) mixture instead of the extract.

#### 3.7.3. DPPH and ABTS Methods

The antioxidant activity by the DPPH method was determined following the protocol by Lisiecka and Wójtowicz [64]. To assess the antioxidant activity, 2.5 mL of a freshly prepared 0.2 mM/L solution of 2,2-diphenyl-1-picrylhydrazyl (DPPH) in methanol was mixed with 0.1 mL of the extract. After 30-min incubation in a dark environment, the decrease in absorbance induced by the sample was measured at 515 nm. The absorbance of the DPPH solution was 0.7 ± 0.05.

The antioxidant activity using the ABTS method was conducted in accordance with the modified protocol by Re et al. [65]. ABTS was dissolved to achieve a concentration of 7 mM in water. It was then mixed with potassium persulfate to reach a final concentration of 2.45 mM, generating ABTS+ radicals. The mixture was left in the dark at room temperature for 12 h before use. The resulting solution was diluted with distilled water to obtain an absorbance of 0.7 ± 0.02 at a wavelength of 734 nm. Subsequently, 0.6 mL of the extract was added to 2.7 mL of the ABTS+ solution, and the absorbance was measured after 15 min. 

Measurements of TPC and AA were performed using the Spectrophotometer Model 9423 (Alt, East Lyme, CT, USA). The blank samples comprised methanol:water (1:1, *v*/*v*) mixture in place of the extract. 

The radical scavenging activity was determined according to the following formula [66]:

(11) $$\%Inhibition=\left(\frac{A_{blank}-A_{sample}}{A_{blank}}\right)\times 100$$

where A_blank_ is the absorption of a blank sample, and A_sample_ is the absorption of a tested sample with DPPH/ABTS reagent. The percentage of DPPH and ABTS inhibition is presented in Figures S1 and S2 (Supplementary Material).

The values for half-maximal inhibitory concentration (EC_50_) were determined by calculating the concentration of the PP extract at which 50% of the maximum inhibition was achieved, as indicated by the fitted models employing a dose-dependent mode of action [67].

### 3.8. Quantitative Analysis of Phytochemicals by LC–MS/MS

#### 3.8.1. Preparation of Extracts 

One gram of powdered PP underwent extraction using 10 mL of methanol in an ultrasonic bath for a duration of 3 h at 40 °C with a frequency of 28 kHz. These extraction conditions were selected based on prior experimentation. Subsequently, the tubes containing the extracted material underwent centrifugation. The resulting upper layer was then filtered through a 0.2 µm string filter.

#### 3.8.2. Test Solution for Mass Spectrometer and Chromatography Conditions

The study outlined the analytical methodology employed was elucidated by Yilmaz [68]. A Shimadzu–Nexera ultrahigh performance liquid chromatograph (UHPLC) coupled with a tandem mass spectrometer (USA, Columbia) was employed for the quantitative analysis of 53 phytochemicals. The UHPLC system, comprising an autosampler (SIL-30AC), a column oven (CTO-10ASvp), binary pumps (LC-30AD), and a degasser (DGU-20A3R) was utilized. The optimization of chromatographic conditions aimed to achieve the optimal separation of the 53 phytochemicals and mitigate suppression effects. Various columns, such as Agilent Poroshell 120 EC-C18 (150 mm × 2.1 mm, 2.7 µm) and RP-C18 Inertsil ODS-4 (100 mm × 2.1 mm, 2 µm), along with diverse mobile phases (B) including acetonitrile and methanol, and different mobile phase additives (ammonium formate, formic acid, ammonium acetate, and acetic acid) were investigated. Additionally, different column temperatures ranging from 25 °C to 40 °C were examined. The optimal chromatographic separation was achieved using an Agilent Poroshell 120 EC-C18 column (150 mm × 2.1 mm, 2.7 µm) at 40 °C. Eluent A (water + 5 mM ammonium formate + 0.1% formic acid) and eluent B (methanol + 5 mM ammonium formate + 0.1% formic acid) were used to create an elution gradient with the following profiles: 20–100% B (0–25 min), 100% B (25–35 min), and 20% B (35–45 min). The injection volume and flow rate of the solvent were set at 0.5 mL/min and 5 L, respectively. The Shimadzu LCMS-8040 tandem mass spectrometer, equipped with electrospray ionization (ESI) source operating in both negative and positive ionization modes, was employed for mass spectrometric detection. The LC-ESI-MS/MS data were processed using LabSolutions software (version 5.97, Shimadzu). The MRM (multiple reaction monitoring) mode was optimized to selectively detect and quantify phytochemical compounds based on specified precursor-to-fragment ion transitions. Collision energies (CE) were optimized to achieve optimal phytochemical fragmentation and maximal transmission of desired product ions. The MS operating conditions included a drying gas (N2) flow of 15 L/min, nebulizing gas (N2) flow of 3 L/min, DL temperature at 250 °C, heat block temperature at 400 °C, and interface temperature at 350 °C.

Since the LC-MS/MS method utilized had been previously developed and validated, comprehensive information regarding the method’s validation and development can be found in the study by Yilmaz, 2020 [68].

### 3.9. Statistical Analysis of Data

The analysis was conducted in triplicate, and the acquired test results underwent statistical analysis, involving the calculation of mean values and standard deviations. Subsequently, a one-way analysis of variance was performed, and the Tukey test was employed to assess the significance of differences between the means. The regression analysis was used to evaluate the drying kinetics of PP, and the coefficient of determination (R2), mean-square error (RMSE), and Chi-quadrate test (χ^2^) were calculated [69]. The statistical analysis was conducted utilizing Statistica 14.0 software (StatSoft, Inc., Tulsa, OK, USA), with a significance level of α = 0.05 considered as an indicator of statistical importance.

## 4. Conclusions

Contact-drying of PP at 40 °C assisted by microwaves proved to be the fastest among the selected drying methods. Additionally, freeze-drying exhibited a shorter duration compared to the contact-drying method at the same temperature of 60 °C. Lyophilized PP, being more susceptible to comminution, required less energy input for size reduction compared to contact-dried pomace. Furthermore, freeze-dried PP displayed a brighter, less yellow, and less red coloration than the contact-dried raw material. 

The lyophilization of PP resulted in an increase in TPC and improved AA compared to contact-drying, whether or not microwave assistance was applied. The elevation of the contact-drying temperature and the use of microwave assistance during contact-drying positively influenced the TPC and AA of PP.

In terms of specific phenolic components, PP dried through contact-drying at 60 °C showed higher levels of quinic and protocatechuic acids compared to lyophilized pomace at the same temperature. Conversely, lyophilized pomace exhibited increased concentrations of chlorogenic acid, epicatechin, and catechin. The content of certain phenolic components of PP, such as gallic acid and epicatechin, varied depending on the applied drying temperature.

Taking into consideration both TPC and AA, the most favorable PP powder was obtained through freeze-drying at a temperature of 20 °C.

## Figures and Tables

**Table 1:** Analysis of models describing the contact-drying process of PP.

Model Name	Sample								
	40 °C+M			60 °C			80 °C		
	R^2^	RMSE	χ^2^	R^2^	RMSE	χ^2^	R^2^	RMSE	χ^2^
Newton	0.943	0.033	1.137 × 10^−3^	0.996	0.003	1.002 × 10^−5^	0.983	0.004	1.229 × 10^−4^
Page	0.988	0.007	5.006 × 10^−5^	0.999	0.000	5.052 × 10^−8^	0.998	0.002	2.924 × 10^−6^
Handerson and Pabis	0.968	0.019	3.658 × 10^−4^	0.999	0.006	4.032 × 10^−5^	0.994	0.004	1.525 × 10^−5^
Logarithmic	0.999	0.000	1.084 × 10^−7^	0.996	0.003	8.597 × 10^−6^	0.972	0.017	2.880 × 10^−4^
Wang and Singh	0.972	0.017	2.958 × 10^−4^	0.997	0.002	2.469 × 10^−6^	0.995	0.030	1.341 × 10^−5^
Logistic	0.983	0.004	7.557 × 10^−6^	0.999	0.000	3.789 × 10^−7^	0.998	0.002	3.172 × 10^−6^
Midilli	1.000	0.000	7.513 × 10^−9^	1.000	0.000	1.187 × 10^−7^	0.999	0.000	1.149 × 10^−7^

M—microwaves 50 W; R^2^—the coefficient of determination; RMSE—mean-square error; χ^2^—chi-quadrate test.

**Table 2:** Analysis of models describing the freeze-drying process of PP.

Model Name	Sample								
	20 °C			40 °C			60 °C		
	R^2^	RMSE	χ^2^	R^2^	RMSE	χ^2^	R^2^	RMSE	χ^2^
Newton	0.974	0.020	3.889 × 10^−4^	0.926	0.054	2.961 × 10^−3^	0.942	0.032	9.958 × 10^−4^
Page	0.999	0.004	1.578 × 10^−5^	0.999	0.010	1.060 × 10^−4^	0.995	0.003	7.565 × 10^−6^
Handerson and Pabis	0.988	0.027	7.482 × 10^−4^	0.962	0.058	3.371 × 10^−3^	0.960	0.046	2.130 × 10^−3^
Logarithmic	0.996	0.007	4.794 × 10^−5^	0.985	0.023	5.501 × 10^−4^	0.997	0.004	1.513 × 10^−5^
Wang and Singh	0.995	0.004	1.487 × 10^−5^	0.975	0.018	3.372 × 10^−4^	0.996	0.002	4.253 × 10^−6^
Logistic	0.999	0.000	8.320 × 10^−7^	0.998	0.003	8.061 × 10^−6^	0.998	0.002	5.640 × 10^−6^
Midilli	1.000	0.000	2.403 × 10^−7^	1.000	0.000	1.168 × 10^−7^	0.999	0.001	5.558 × 10^−6^

R^2^—the coefficient of determination; RMSE—mean-square error; χ2—chi-quadrate test.

**Table 3:** Values of parameters in the models describing contact-drying process of PP.

Sample	Equation	Coefficient			
		a	k (min^−1^)	n	b
40 °C+M	Newton		0.016941		
	Page		0.001803	1.542794	
	Handerson and Pabis	1.157736	0.019553		
	Logarithmic	1.211390	0.016550		−0.078593
	Wang and Singh	−0.013401			0.000048
	Logistic	0.340762	0.035442		1.413407
	Midilli	0.999961	0.000641	1.837847	0.000620
60 °C	Newton		0.005523		
	Page		0.003256	1.100223	
	Handerson and Pabis	1.045379	0.005796		
	Logarithmic	1.065026	0.005396		−0.030342
	Wang and Singh	−0.004464			0.000006
	Logistic	3.852251	0.006480		4.972726
	Midilli	1.013219	0.003485	1.093202	0.000022
80 °C	Newton		0.006486		
	Page		0.002026	1.228994	
	Handerson and Pabis	1.094512	0.007148		
	Logarithmic	1.133582	0.006309		−0.057525
	Wang and Singh	−0.005219			0.000008
	Logistic	1.487340	0.009111		2.609647
	Midilli	1.013469	0.001536	1.306291	0.000162

M—microwaves 50 W; k—drying coefficient (min^−1^); a, b—coefficients of the equations; n—exponent.

**Table 4:** Values of parameters in the models describing freeze-drying process of pear pomace.

Sample	Equation	Coefficient			
		a	k (min^−1^)	n	b
20 °C	Newton		0.006719		
	Page		0.001274	1.327668	
	Handerson and Pabis	1.614205	0.007506		
	Logarithmic	1.752144	0.005701		−0.196027
	Wang and Singh	−0.005165			0.000007
	Logistic	1.677831	0.011197		1.730994
	Midilli	1.006016	0.001192	1.348115	0.000048
40 °C	Newton		0.009372		
	Page		0.000376	1.687183	
	Handerson and Pabis	1.711976	0.011112		
	Logarithmic	2.170170	0.006206		−0.556726
	Wang and Singh	−0.006701			0.000010
	Logistic	0.211186	0.022794		1.258798
	Midilli	1.004994	0.000275	1.772532	0.000189
60 °C	Newton		0.010671		
	Page		0.000935	1.538620	
	Handerson and Pabis	1.611008	0.011998		
	Logarithmic	3.14173	0.00370		−1.652820
	Wang and Singh	−0.000109			−0.000038
	Logistic	0.193015	0.025674		1.166118
	Midilli	0.979450	0.001176	1.433943	−0.000678

k—drying coefficient; a, b—coefficients of the equations; n—exponent.

**Table 5:** Parameters describing the particle size distribution of PP dried under different conditions.

DM	DC	SSA	d_10_ (µm)	d_50_ (µm)	d_90_ (µm)	Span
Contact-drying	40 °C+M	34.2 ± 0.13 ^a^	69.0 ± 3.83 ^e^	251.7 ± 10.79 ^a^	520.0 ± 24.27 ^a^	1.8 ± 0.01 ^a^
	60 °C	34.1 ± 0.23 ^a^	63.9 ± 0.72 ^d^	245.3 ± 5.83 ^a^	550.7 ± 23.29 ^a^	2.0 ± 0.09 ^ab^
	80 °C	40.8 ± 1.73 ^b^	54.1 ± 0.98 ^a^	236.0 ± 16.82 ^a^	559.7 ± 44.77 ^a^	2.1 ± 0.05 ^b^
Freeze-drying	20 °C	44.7 ± 1.38 ^c^	52.0 ± 0.66 ^a^	159.0 ± 5.00 ^b^	350.0 ± 13.53 ^bc^	1.9 ± 0.02 ^a^
	40 °C	48.3 ± 0.71 ^d^	46.4 ± 0.85 ^c^	184.0 ± 1.73 ^c^	401.6 ± 37.65 ^c^	1.9 ± 0.19 ^ab^
	60 °C	58.4 ± 1.44 ^e^	39.2 ± 0.87 ^b^	140.7 ± 3.06 ^b^	320.33 ± 5.77 ^b^	2.0 ± 0.02 ^ab^

DM—drying method; DC—drying conditions; SSA—specific surface area; d_10_, d_50_, d_90_—represent the 10th, 50th, and 90th percentile of the total volume, presuming the particles exhibit a spherical form; Span—the size dispersion index; M—microwaves 50 W; the values are expressed as mean ± SD; means with different letter superscript are significantly different (α = 0.05).

**Table 6:** Moisture content, water activity, and grinding energy indices of PP.

DM	DC	MC (%)	a_w_	E_s_ (kJ·kg^−1^)	E_f_ (m^2^·kJ^−1^)
Contact-drying	40 °C+M	7.55 ± 0.12 ^d^	0.251 ± 0.004 ^d^	12.13 ± 0.56 ^b^	2.82 ± 0.12 ^a^
	60 °C	7.90 ± 0.08 ^e^	0.275 ± 0.005 ^e^	12.66 ± 0.94 ^b^	2.70 ± 0.22 ^a^
	80 °C	7.56 ± 0.07 ^d^	0.268 ± 0.006 ^e^	12.06 ± 0.53 ^b^	3.39 ± 0.27 ^a^
Freeze-drying	20 °C	6.78 ± 0.04 ^b^	0.238 ± 0.005 ^c^	8.83 ± 0.44 ^a^	5.07 ± 0.38 ^b^
	40 °C	6.52 ± 0.05 ^a^	0.226 ± 0.002 ^b^	8.70 ± 0.65 ^a^	5.58 ± 0.44 ^b^
	60 °C	6.31 ± 0.09 ^a^	0.211 ± 0.003 ^a^	9.07 ± 0.29 ^a^	6.45 ± 0.29 ^c^

DM—drying method, DC—drying conditions, MC—moisture content, a_w_—water activity, E_s_—specific grinding energy, E_f_—grinding efficiency index, M—microwaves 50 W; the values are expressed as mean ± SD; means with different letter superscript are significantly different (α = 0.05).

**Table 7:** Influence of drying method and temperature on the color of PP.

DM	DC	L*	a*	b*	h*
Contact-drying	40 °C+M	62.52 ± 0.24 ^c^	9.30 ± 0.06 ^d^	22.54 ± 0.19 ^c^	67.57 ± 0.05 ^b^
	60 °C	61.56 ± 0.38 ^c^	9.63 ± 0.10 ^d^	22.22 ± 0.24 ^bc^	66.56 ± 0.19 ^a^
	80 °C	58.16 ± 0.47 ^b^	8.82 ± 0.13 ^c^	21.20 ± 0.68 ^b^	67.40 ± 0.36 ^b^
Freeze-drying	20 °C	71.19 ± 0.16 ^d^	7.71 ± 0.14 ^b^	24.41 ± 0.40 ^d^	72.46 ± 0.05 ^d^
	40 °C	70.32 ± 0.16 ^d^	7.92 ± 0.02 ^b^	25.24 ± 0.74 ^d^	71.29 ± 0.57 ^c^
	60 °C	70.22 ± 0.20 ^d^	7.34 ± 0.07 ^a^	24.95 ± 0.20 ^d^	73.61 ± 0.02 ^e^

DM—drying method, DC—drying conditions, L*—lightness, a*—redness, b*—yellowness, h*— hue angle; M—microwaves 50 W; the values are expressed as mean ± SD; means with different letter superscript are significantly different (α = 0.05).

**Table 8:** Antioxidant activity of PP depending on drying method and conditions.

DM	DC	TPC	DPPH (EC_50_)	ABTS (EC_50_)
Contact-drying	40 °C+M	4.05 ± 0.08 ^c^	71.34 ± 2.48 ^b^	58.51 ± 1.01 ^c^
	60 °C	2.58 ± 0.01 ^a^	92.14 ± 4.37 ^c^	81.77 ± 0.38 ^d^
	80 °C	3.46 ± 0.03 ^b^	87.15 ± 1.98 ^c^	68.82 ± 0.47 ^e^
Freeze-drying	20 °C	4.32 ± 0.05 ^d^	43.67 ± 0.35 ^a^	40.78 ± 0.41 ^a^
	40 °C	4.32 ± 0.02 ^d^	44.98 ± 0.27 ^a^	44.21 ± 0.19 ^b^
	60 °C	4.29 ± 0.06 ^d^	44.46 ± 0.43 ^a^	45.13 ± 0.46 ^b^

DM—drying method; DC—drying conditions; TPC—total phenolics content expressed in mg gallic acid equivalent (GAE)·g^−1^ DW; DPPH (EC_50_)—ability to neutralize DPPH radicals expressed as EC_50_ in mg DW·mL^−1^; ABTS (EC_50_)—ability to scavenge ABTS radicals expressed as EC_50_ in mg DW·mL^−1^; M—microwaves 50 W; the values are expressed as mean ± SD; means with different letter superscript are significantly different (α = 0.05).

**Table 9:** Quantitative screening of phytochemicals in powdered PP using LC–MS/MS (mg·g^−1^ DW).

Analytes	Contact-Drying			Freeze-Drying		
	40 °C+M	60 °C	80 °C	20 °C	40 °C	60 °C
Quinic acid	145.04 ± 10.15 ^a^	198.92 ± 9.95 ^b^	189.29 ± 15.14 ^b^	128.75 ± 11.59 ^a^	125.79 ± 6.29 ^a^	132.14 ± 6.61 ^a^
Fumaric acid	nd	nd	nd	nd	nd	nd
Aconitic acid	nd	nd	nd	nd	nd	nd
Gallic acid	nd	1.30 ± 0.08 ^b^	0.08 ± 0.01 ^a^	4.74 ± 0.47 ^d^	1.97 ± 0.12 ^c^	2.22 ± 0.20 ^c^
Epigallocatechin	nd	nd	nd	nd	nd	nd
Protocatechuic acid	1.06 ± 0.06 ^a^	4.21 ± 0.46 ^b^	1.64 ± 0.08 ^a^	3.75 ± 0.34 ^b^	1.69 ± 0.10 ^a^	1.65 ± 0.10 ^a^
Catechin	nd	nd	nd	0.43 ± 0.03 ^b^	0.34 ± 0.02 ^ab^	0.30 ± 0.02 ^a^
Gentisic acid	nd	nd	nd	nd	nd	nd
Chlorogenic acid	2.47 ± 0.15 ^b^	0.29 ± 0.01 ^a^	1.50 ± 0.11 ^b^	5.98 ± 0.66 ^d^	4.54 ± 0.41 ^c^	6.36 ± 0.64 ^d^
Protocatechuic aldehyde	0.170 ± 0.010 ^b^	0.067 ± 0.003 ^a^	0.165 ± 0.012 ^b^	0.255 ± 0.023 ^d^	0.221 ± 0.018 ^cd^	0.191 ± 0.011 ^bc^
Tannic acid	nd	nd	nd	nd	nd	nd
Epigallocatechin gallate	nd	nd	nd	nd	nd	nd
Cynarin	nd	nd	nd	nd	nd	nd
4-OH Benzoic acid	nd	nd	nd	nd	nd	nd
Epicatechin	nd	nd	nd	15.34 ± 1.53 ^a^	13.78 ± 1.24 ^a^	21.59 ± 2.37 ^b^
Vanillic acid	nd	nd	nd	nd	nd	nd
Caffeic acid	0.041 ± 0.002 ^b^	0.021 ± 0.001 ^a^	0.04 ± 0.003 ^b^	0.05 ± 0.004 ^b^	0.046 ± 0.004 ^b^	0.101 ± 0.009 ^d^
Syringic acid	nd	nd	nd	nd	nd	nd
Vanillin	0.563 ± 0.021 ^d^	0.248 ± 0.012 ^b^	0.502 ± 0.035 ^c^	0.255 ± 0.013 ^b^	0.101 ± 0.005 ^a^	0.089 ± 0.004 ^a^
Syringic aldehyde	0.183 ± 0.005 ^c^	0.107 ± 0.005 ^b^	0.23 ± 0.016 ^d^	nd	0.074 ± 0.004 ^a^	0.086 ± 0.007 ^ab^
Daidzin	nd	nd	nd	nd	nd	nd
Epicatechin gallate	nd	nd	nd	nd	nd	nd
Piceid	nd	nd	nd	nd	nd	nd
p-Coumaric acid	nd	nd	nd	nd	nd	nd
Ferulic acid-D3-IS^h^	N.A.	N.A.	N.A.	N.A.	N.A.	N.A.
Ferulic acid	nd	nd	nd	nd	nd	nd
Sinapic acid	nd	nd	nd	nd	nd	nd
Coumarin	nd	nd	nd	nd	nd	nd
Salicylic acid	0.0080 ± 0.0003 ^a^	0.0110 ± 0.0009 ^a^	0.0650± 0.0072 ^b^	nd	nd	nd
Cyranoside	nd	nd	nd	nd	nd	nd
Miquelianin	nd	nd	nd	nd	nd	nd
Rutin-D3-IS	na	na	na	na	na	na
Rutin	1.71 ± 0.17 ^b^	0.92 ± 0.07 ^a^	2.67 ± 0.29 ^c^	1.83 ± 0.16 ^b^	1.43 ± 0.09 ^b^	1.66 ± 0.12 ^b^
Isoquercitrin	8.27 ± 0.74 ^b^	4.03 ± 0.24 ^a^	12.58 ± 0.88 ^c^	13.89 ± 0.69 ^c^	8.48 ± 0.59 ^b^	9.92 ± 0.60 ^b^
Hesperidin	0.87 ± 0.06 ^bc^	0.49 ± 0.02 ^a^	1.25 ± 0.13 ^d^	0.97 ± 0.05 ^c^	0.75 ± 0.05 ^b^	0.95 ± 0.08 ^c^
o-Coumaric acid	nd	nd	nd	nd	nd	nd
Genistin	nd	nd	nd	nd	nd	nd
Rosmarinic acid	nd	nd	nd	nd	nd	nd
Ellagic acid	nd	nd	nd	nd	nd	nd
Cosmosiin	nd	nd	nd	nd	nd	nd
Quercitrin	nd	nd	nd	nd	nd	nd
Astragalin	0.203 ± 0.008 ^b^	0.121 ± 0.007 ^a^	0.391 ± 0.023 ^d^	0.337 ± 0.030 ^c^	0.209 ± 0.017 ^b^	0.236 ± 0.010 ^b^
Nicotiflorin	0.28 ± 0.02 ^a^	0.22 ± 0.02 ^a^	0.55 ± 0.06 ^b^	0.29 ± 0.01 ^a^	0.24 ± 0.01 ^a^	0.26 ± 0.02 ^a^
Fisetin	nd	nd	nd	nd	nd	nd
Daidzein	nd	nd	nd	nd	nd	nd
Quercetin-D3-IS	N.A.	N.A.	N.A.	N.A.	N.A.	N.A.
Quercetin	0.442 ± 0.040 ^d^	0.126 ± 0.004 ^b^	0.368 ± 0.029 ^c^	0.07 ± 0.004 ^a^	0.061 ± 0.005 ^a^	0.079 ± 0.006 ^ab^
Naringenin	0.0120 ± 0.0006 ^d^	0.0090 ± 0.0005 ^c^	0.0220 ± 0.0002 ^e^	0.0050 ± 0.0003 ^a^	0.0070 ± 0.0004 ^b^	0.0060 ± 0.0005 ^ab^
Hesperetin	nd	nd	nd	nd	nd	nd
Luteolin	nd	nd	nd	nd	nd	nd
Genistein	nd	nd	nd	nd	nd	nd
Kaempferol	0.0180 ± 0.0009 ^b^	0.0110 ± 0.0007 ^a^	0.0240 ± 0.0019 ^c^	nd	nd	nd
Apigenin	nd	nd	nd	nd	nd	nd
Amentoflavone	nd	nd	nd	nd	nd	nd
Chrysin	nd	nd	0.0033 ± 0.0001 ^a^	0.0040 ± 0.0002 ^b^	nd	0.0030 ± 0.0002 ^a^
Acacetin	0.0040 ± 0.0002 ^b^	nd	0.0030 ± 0.0002 ^a^	nd	nd	nd

M—microwaves 50 W; nd—not detected, N.A.—not applicable; the values are expressed as mean ± SD; means with different letter superscript are significantly different (α = 0.05).

**Table 10:** Mathematical models for drying curve analysis.

Model Name	Equation
Handerson and Pabis [52]	(2) $MR=a\cdot \exp\left(-k\cdot \tau \right)$
Logarithmic [53]	(3) $MR=a\cdot \exp\left(-k\cdot \tau \right)+b$
Logistic [54]	(4) MR=b·((1+a·exp⁡(k·τ))−1
Midilli [55]	(5) $MR=a\cdot \exp\left(-k\cdot \tau^{n}\right)+b\cdot \tau$
Newton [56]	(6) MR=exp⁡(k·τ)
Page [57]	(7) $MR=exp\left(-k\cdot \tau^{n}\right)$
Wang and Singh [58]	(8) $MR=1+a\cdot \tau +b\cdot \tau^{2}\;$

k—drying coefficient (min^−1^); a, b—coefficients of the equations; n—exponent; τ—time (min).

## Data Availability

The data presented in this study are available on request from the corresponding author.

## Supplementary Materials

The following supporting information can be downloaded at: https://www.mdpi.com/article/10.3390/molecules29030742/s1, Figure S1: Inhibition of DPPH by powdered pear pomace; Figure S2: Inhibition of ABTS by powdered pear pomace.
