# Supplementary material for: Physicochemical Properties of Dried and Powdered Pear Pomace
Source: Molecules. 2024 Feb 5;29(3):742. doi: 10.3390/molecules29030742 (PMC10856639; doi:10.3390/molecules29030742)
Supplement: Supplementary file 1 [file molecules-29-00742-s001.zip › molecules-2848984-supplementary.pdf]

Supplementary Material

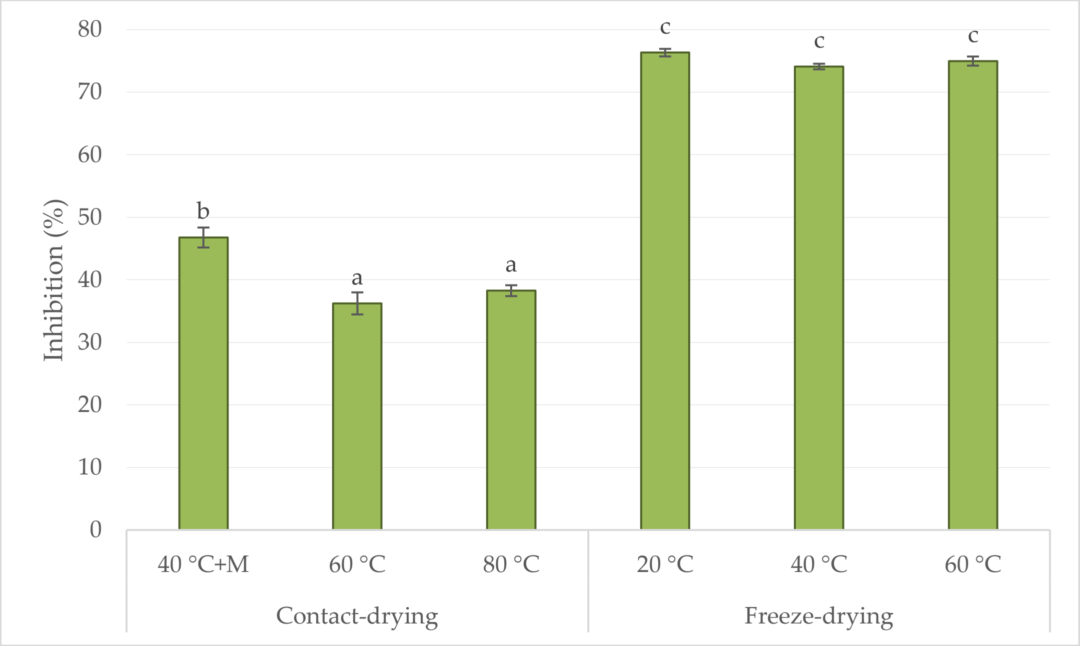

Figure S1. Inhibition of DPPH by powdered pear pomace.

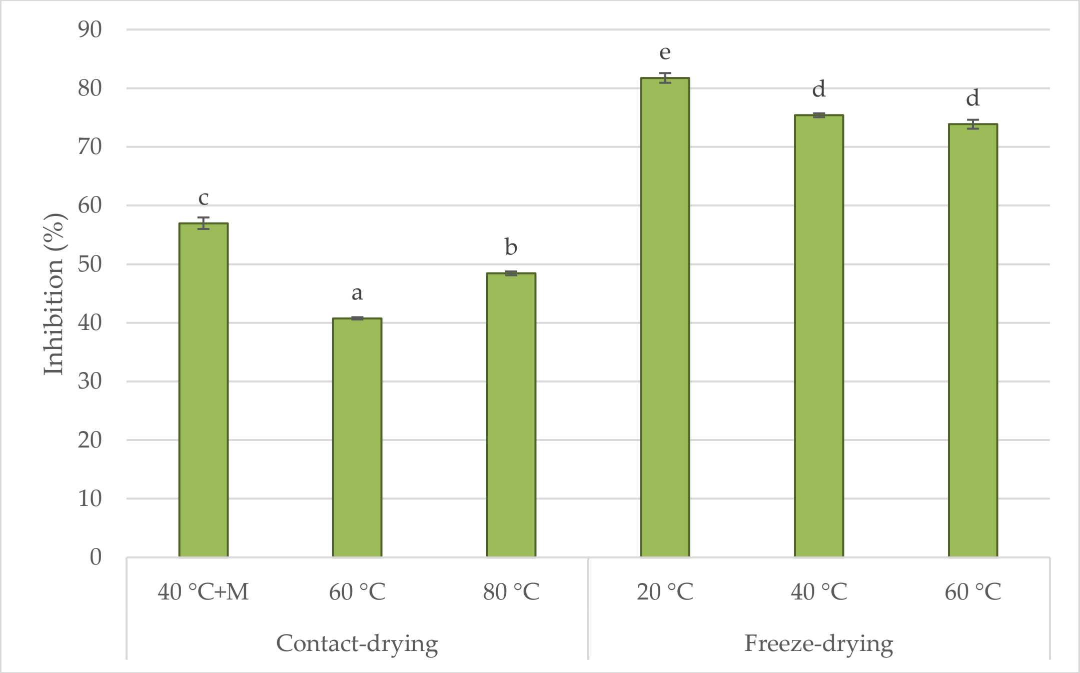

Figure S2. Inhibition of ABTS by powdered pear pomace.
